# In Vivo Follow-up of Brain Tumor Growth via Bioluminescence Imaging and Fluorescence Tomography

**DOI:** 10.3390/ijms17111815

**Published:** 2016-10-31

**Authors:** Coralie Genevois, Hugues Loiseau, Franck Couillaud

**Affiliations:** Molecular Imaging and Innovative Therapy in Oncology (IMOTION), EA 7435, University of Bordeaux, Bordeaux 33076, France; coralie.genevois@u-bordeaux.fr (C.G.); hugues.loiseau@chu-bordeaux.fr (H.L.)

**Keywords:** reporter gene, optical imaging, bioluminescence, fluorescence tomography, cancer, glioblastoma

## Abstract

Reporter gene-based strategies are widely used in experimental oncology. Bioluminescence imaging (BLI) using the firefly luciferase (Fluc) as a reporter gene and d-luciferin as a substrate is currently the most widely employed technique. The present paper compares the performances of BLI imaging with fluorescence imaging using the near infrared fluorescent protein (iRFP) to monitor brain tumor growth in mice. Fluorescence imaging includes fluorescence reflectance imaging (FRI), fluorescence diffuse ^optical^ tomography (fDOT), and fluorescence molecular Imaging (FMT^®^). A U87 cell line was genetically modified for constitutive expression of both the encoding Fluc and iRFP reporter genes and assayed for cell, subcutaneous tumor and brain tumor imaging. On cultured cells, BLI was more sensitive than FRI; in vivo, tumors were first detected by BLI. Fluorescence of iRFP provided convenient tools such as flux cytometry, direct detection of the fluorescent protein on histological slices, and fluorescent tomography that allowed for 3D localization and absolute quantification of the fluorescent signal in brain tumors.

## 1. Introduction

Reporter gene-based technologies are powerful tools for modern biomedical research. Fluorescent proteins and luciferases have long been used for in vitro applications in molecular and cellular biology. Optical imaging reporters are also effective for in vivo imaging in preclinical studies. The most commonly used imaging reporter gene is the encoding firefly luciferase (Fluc), which catalyzes d-luciferin oxidation to produce photons that can be detected in vivo by whole body bioluminescence imaging (BLI) [1,2]. BLI is cost-effective and allows for the detection of minor events because of its low background and high sensitivity. The enzymatic reaction requires co-factors such as Mg^2+^, adenosine triphosphate (ATP), and oxygen. The fast rate of Fluc turnover in the presence of the d-luciferin substrate allows for real-time measurements, and the relationship between Fluc concentration and photon emission in vitro is linear. Although widely used, in vivo BLI has several limitations. It is essentially a two-dimensional (2D) imaging technique making a deep tumor localization inexact. The bioluminescence signal is dependent on several factors, such as d-luciferin distribution [3], co-factor availability, signal depth, and tissue absorption; as such, the absolute quantification of photons is usually not accessible. Furthermore, regarding upstream and downstream in vitro processes, Fluc-expressing cells are not detectable by flux cytometry, and subcellular detection of Fluc requires antibody-based procedures. 

Fluorescence tomography techniques coupled with fluorescent reporter proteins offer several advantages compared to BLI. Fluorescence tomography is a three-dimensional (3D) technique that enables tumor localization in deep tissues and absolute quantification of the fluorescence signal [4]. The most common fluorescent protein is the green fluorescent protein (GFP), but its excitation and emission wavelengths are not optimal for deeper in vivo imaging because of absorption and tissue autofluorescence. To overcome these limitations, fluorescent proteins with excitation and emission spectra within the near infrared (NIR) window must be used. Fluorescent tomography systems have been built to fit with tissue absorption requirements and are operating in the NIR windows [4,5], but choice and performance of fluorescent reporter proteins for in vivo use is currently limited. Quite recently, a near infrared fluorescent protein, infrared fluorescent protein (iRFP), was described [6], and several mutated forms were obtained [7]. The original iRFP, namely iRFP713, is a fluorescent mutant of a bacteriophytochrome from *Rhodopseudomonas palustris* whose excitation and emission wavelengths are 690 and 713 nm, respectively. iRFP is a relatively stable and bright dimer protein of 190 Kd [6]. The iRFP fluorescence signal is due to direct detection of the protein dimer and does not require any exogenous co-factors to emit fluorescence.

The present paper investigates the performance of iRFP for in vivo imaging experimentation and compares Fluc/BLI with iRFP/fluorescence tomography to monitor brain tumor growth in mice.

## 2. Results

### 2.1. In Vitro Correlation between Fluc Activity and Infrared Fluorescent Protein (iRFP) Expression

**Figure 1:**
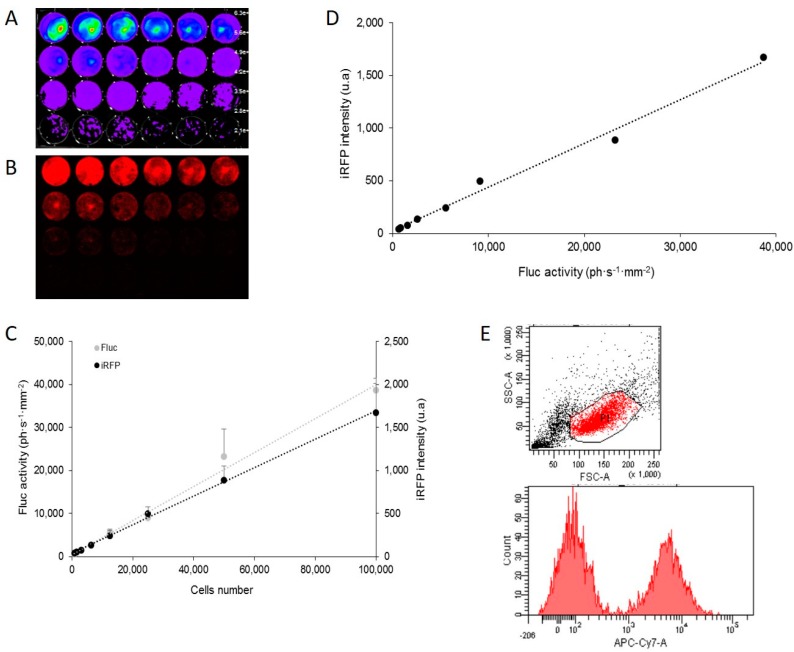
In vitro correlation between firefly luciferase (Fluc) activity and infrared fluorescent protein (iRFP) expression in U87-iRFP+-Fluc+ cells. Bioluminescence imaging (BLI) (**A**) and fluorescence reflectance imaging (FRI) (**B**) of successive dilutions (1:2) of U87-iRFP+-Fluc+ cells (from 781 to 100,000 cells) two days after plating. Fluorescent signals of iRFP and Fluc activities were plotted versus cell number (**C**). Fluorescent signals of iRFP were plotted versus bioluminescence signals (**D**). Representative distribution of iRFP-expressing cells (**E**) was determined by flux cytometry (10,000 cells). u.a, units arbitrary.

A bicistronic vector has been constructed for cap-dependent translation of iRFP and internal ribosome entry site (IRES)-dependent translation of Fluc, both reporters being under transcriptional control of a unique constitutive CMV promoter. This vector was introduced in the U87 cell line to compare iRFP with Fluc expression. The U87-FRT-CMV-iRFP-IRES-Fluc cell line (U87-iRFP+-Fluc+) was first assayed for Fluc enzymatic activity in vitro on cell lysate. Fluc activity resulting from IRES-dependent translation was found to be 4.9 × 10^6^ RLU per 100-cell equivalent, quite similar to the Fluc activity of U87-FRT-CMV-Fluc cell line (5 × 10^6^ RLU per 100-cell equivalent) resulting from cap-dependent translation as previously published [8]. In the in vitro study, different quantities of cells (ranging from 781 to 100,000) were plated, and expression of the two reporter genes was detected with imaging on living cells 48 h later via BLI for Fluc (Figure 1A) and fluorescence scanning for iRFP (Figure 1B). Quantification of optical signals revealed a strong correlation between the number of cells plated and Fluc activity (*r*^2^ = 0.9986) or iRFP fluorescence (*r*^2^ = 0.9905) (Figure 1C). iRFP fluorescence and Fluc activity were also closely correlated (*r*^2^ = 0.9906, Figure 1D). Altogether, these data showed that the two reporter genes could be used in U87 cells and allowed for cell quantification in vitro. BLI sensitivity was higher than NIR fluorescence. As shown in Figure 1E, iRFP could also be detected by the flux cytometry of U87 cells.

### 2.2. Monitoring Subcutaneous Tumor Growth by Optical Imaging

**Figure 2:**
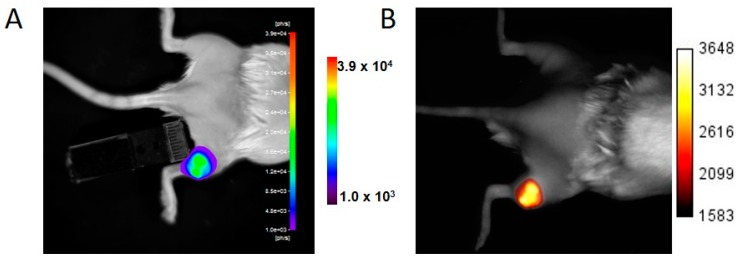

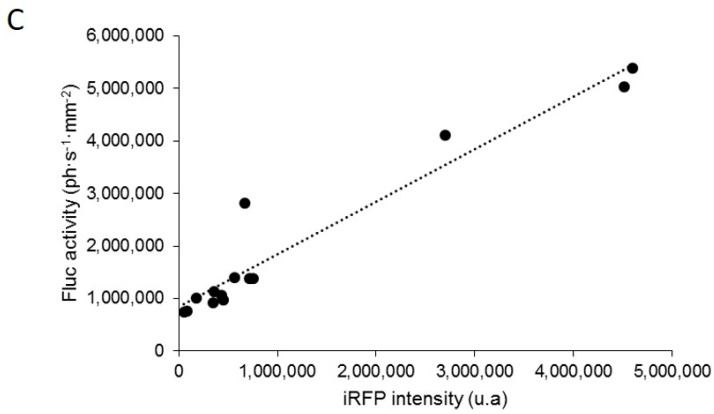
In vivo detection of Fluc activity and iRFP fluorescence by subcutaneous tumors U87-iRFP+-Fluc+. BLI (**A**) and FRI (**B**) of a representative mouse. Bioluminescence signals were plotted versus iRFP fluorescence signals (**C**) (*n* = 8).

U87-iRFP+-Fluc+ cells (2 × 10^6^ cells) were injected subcutaneously into the posterior right leg of the mice (*n* = 8). Each mouse was then imaged twice—for Fluc activity and iRFP expression—when the tumor diameter reached about 5 and 10 mm, respectively. Fluc activity (Figure 2A) and iRFP fluorescence (Figure 2B) were detected by BLI and FRI, respectively, for both tumor sizes. Quantification of optical signals revealed a good correlation (*r*^2^ = 0.9282) between Fluc activity and iRFP fluorescence (Figure 2C).

### 2.3. Monitoring Brain Tumor Growth with Optical Imaging

**Figure 3:**
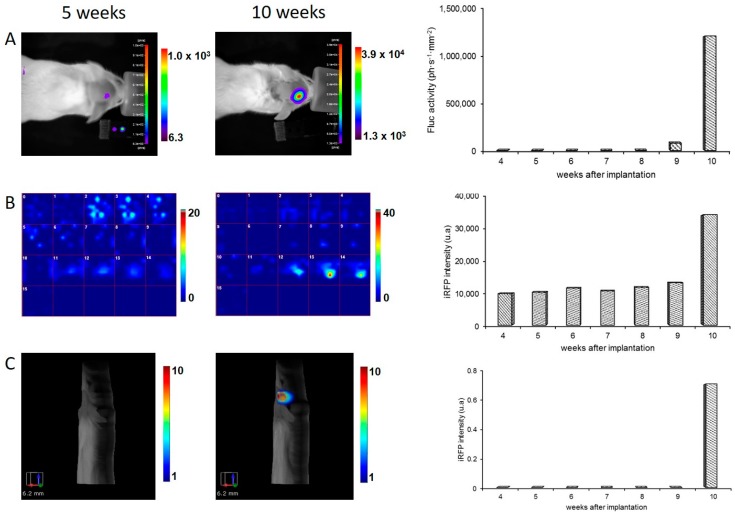
In vivo detection of Fluc activity and iRFP fluorescence by brain tumors U87-iRFP+-Fluc+. (**A**) BLI of a mouse at 5 weeks and at 10 weeks after cell injection. The graph represents the bioluminescence quantification of the mouse; (**B**) detection of the iRFP-fluorescent signal by fluorescence diffuse optical tomography (fDOT) of the same mouse at 5 and 10 weeks after cell injection. Z cross sections (1 mm thickness) are presented in the same color scale from z = 0 (ventral) to z = 15 (dorsal). Quantification of the fluorescence signal recovered from fDOT imaging at different times are plotted on the graph; (**C**) 3D representation of the iRFP-fluorescent signal by FMT^®^ of a mouse at 5 and 10 weeks after cell injection. Quantification of the fluorescence signal recovered from FMT^®^ imaging at different times are plotted on the graph.

Two series of mice received a stereotaxic injection of U87-iRFP+-Fluc+ cells (6 × 10^5^ or 1 × 10^6^ cells) to generate deep tumors in the brain. Brain tumor growth was monitored four weeks after cell injection by BLI and fluorescence tomography over several weeks using different imaging devices. For the first batch of injected mice (*n* = 8), Fluc activity was detected by BLI, and iRFP fluorescence was followed by fluorescence diffuse optical tomography (fDOT). In Figure 3A,B, the mouse was imaged from Week 4 to Week 10. BLI analysis (Figure 3A) showed that Fluc activity was detected as early as Week 5 and that a strong signal was observed in Week 10. Quantification of the BLI signal is reported on the graph and shows a rather constant signal from Week 4 to Week 8. The signal increased in Week 9 and was even stronger in Week 10. As shown in Figure 3B, a fluorescence map was reported as a series of 16 z slices of 1 mm thickness. Whereas Fluc activity was detectable early, a high background fluorescence signal was found in Week 5, making the tumor undetectable. In Week 10, a strong fluorescent signal was detected in Slices 12, 13, and 14, corresponding to the location of the tumor in the brain. Quantification of the fluorescence signals is represented on the graph illustrating a high background fluorescent signal up to Week 9, making the tumor detectable only up from Week 10. The same BLI pattern was observed in the other mice of this experimental series (*n* = 8). The BLI signal remained stable for several weeks and then increased rapidly up to about 10^6^ photons·s^−1^·cm^−2^ between Weeks 10 and 12. The iRFP signal became detectable at that moment. These data showed that we can detect iRFP fluorescence only when Fluc activity is strong.

The second batch of brain-injected mice (*n* = 4) was followed by BLI and fluorescence molecular tomography (FMT^®^). The BLI pattern is almost identical to the previous series with a low BLI signal detected from Week 5 and a rapid increase occurring in Weeks 10 to 13 (data not shown). As illustrated in Figure 3C, the fluorescent signal of iRFP is not discernable from the background fluorescent signal by FMT^®^ at Week 5. In Week 10, when the BLI signal reached 10^6^ photons·s^−1^·cm^−2^, the fluorescent signal was detectable allowing for 3D reconstruction of the brain tumor. Quantification of the fluorescent signal within the tumor volume was determined by FMT^®^ software, and the time course is represented on the graph.

### 2.4. Ex Vivo Imaging and iRFP Histology

**Figure 4:**
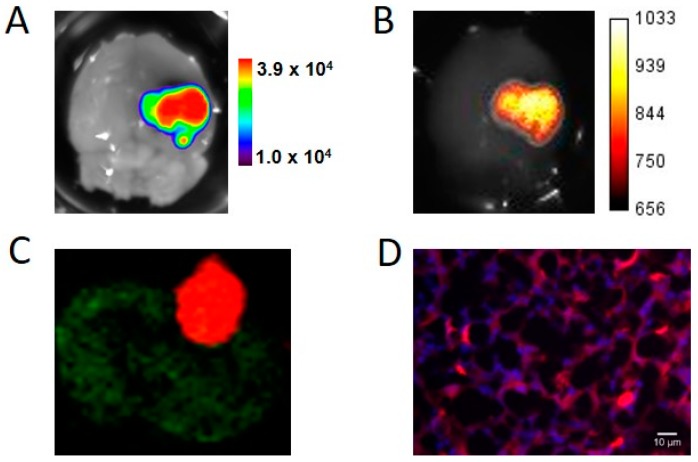
Ex vivo imaging and histology of U87-iRFP+-Fluc+ brain tumors. An excised brain was sequentially imaged by BLI (**A**) and FRI (**B**) to reveal tumors; (**C**) Cryosection of the brain tumor was scanned for fluorescence using the Odyssey scanner showing iRFP fluorescence at 700 nm (red) and brain autofluorescence at 800 nm (green); (**D**) Fluorescence signals of iRFP (red) and nucleus (DAPI, blue) were revealed using epifluorescence microscopy.

Mice were euthanized between Weeks 10 and 13, approximately 20 min after d-luciferin injection. Brains were quickly removed and sequentially imaged by BLI for Fluc activity and FRI for iRFP fluorescence. Bioluminescence and fluorescence images clearly showed U87-iRFP+-Fluc+ tumors and showed good correspondence for tumor shape (Figure 4A,B). Tumors were then fixed and frozen. As shown in Figure 4C,D, fluorescence from iRPP could be detected in U87-iRFP+-Fluc+ tumor cells using the Odyssey scanner or epifluorescence microscopy, respectively.

## 3. Discussion

Light absorption by tissues, photon scattering, and autofluorescence are major phenomena affecting in vivo optical imaging. NIR fluorescence tomography overcomes these limitations, making exploration of deep fluorescent signals feasible in mice through the measurement of surface emitted light for the mathematical reconstruction of the source of light emission. Fluorescence tomography thus results from a complex, multi-step process using light wavelengths in the NIR windows, multiple acquisitions of the fluorescent signal with different geometry, tissue absorption measurement, and complex reconstruction algorithms to determine both absorption and fluorescent maps. Many chemical fluorophores are now available that are compatible with NIR tomographs, enabling a large range of imaging strategies in oncology, including those based on the use of blood pool agents, specific probes, or endogenous enzyme-activable probes [9]. For reporter gene strategies, efforts to discover and to engineer NIR-fluorescent proteins have resulted in reports on several fluorescent proteins to be used for whole-body imaging using NIR tomographs, including IFP1.4 [10] and iRFP [6,7]. Because they do not require any exogenous factors to become fluorescent, iRFPs are easy to use and are currently the most attractive fluorescent proteins for whole-body NIR fluorescent imaging of deep tumors, such as orthotopic prostate cancer [11,12], melanoma metastases [13], intraperitoneal tumors [14], or lung adenocarcinoma [15]. 

Reporter gene-based strategies are widely used in oncology preclinical studies. BLI using Fluc as a reporter gene and d-luciferin as a substrate is currently the most widely employed technique. The present data show that, on living cells (i.e., without tissue absorption), Fluc/BLI is about 1000 times more sensitive than iRFP/FRI. The fluorescent signal of iRFP, however, is perfectly correlated to the BLI signal and to the number of cells. As illustrated in this paper, iRFP offers specific useful features that are not available with Fluc, such as detection of iRFP-expressing cells by flux cytometry and direct detection of the protein via fluorescence histology. 

For whole body imaging, emitted light resulting from oxidation of d-luciferin by Fluc activity is of dispersed wavelengths, with a maximum emission at 565 nm, outside the NIR window (700–900 nm). Light from d-luciferin/Fluc reaction is thus highly absorbed by tissues. Because of its low background noise and its high sensitivity, planar BLI using Fluc remains the most sensitive optical imaging method, even for deep tumor detection in mice [8,16]. It has been previously reported that the BLI signal of the U87-CMV-Fluc cells could be detected at any time after cell injection [8]. In the present paper, brain tumor detection was assayed four weeks after injection, and light emission was easily detected by BLI. In Week 4, no fluorescent signal in the tumor area was detected using fluorescent tomography. U87 brain tumors were initially slow developing, but growth was rapid between Weeks 9 and 10 as revealed by the rapid increase in BLI signal. The fluorescent signal of iRFP also became detectable in Week 10 using both the fDOT and the FMT^®^ devices. Fluorescence tomography provided 3D images of the fluorescent signal, with a low background within the scanned area, allowing for localization of the tumor in the mouse’s head. It should be noted that, when using fluorescent tomography, the scanned zone is defined by the user; in the present paper, this was limited to the mouse’s head to reduce acquisition time. 

Quantification of the BLI signal measures surface emitted light and thus is dependent on tissue absorption, which is related to tumor depth. For an individual tumor, it has been shown that the BLI signal is correlated to tumor size [17] as long as the tumor is not necrotic. Inter-individual comparison is not possible unless the tumors are growing in the same location (this may be the case after stereotaxic injection for instance). Fluorescence tomography allowed for absolute quantification of the fluorescence signal. As such, a comparison between different tumors and different mice was possible.

In the present context, we show that planar BLI remains the most sensitive method for reporter gene-based approaches. Although less sensitive, fluorescence tomography allowed us to detect iRFP fluorescence, thus enabling 3D localization of tumors and absolute quantification of the signal. By using a bicistronic vector, both Fluc and iRFP could be co-expressed, offering the full set of advantages of the two reporter genes and allowing the combination of imaging techniques.

## 4. Materials and Methods

### 4.1. Animal Handling and Tumor Generation

Animal experiments were performed in agreement with French and European directives on the care and use of animals. Protocols were approved by the local ethics committee in animal experimentation (CEEA 50) under agreement A50120196. Immuno-deficient NOG (NOD/SCID/IL-2Rγnull) mice (6- to 10-weeks-old) were reared at the University of Bordeaux (Bordeaux, France) animal facilities. Animals were maintained under a 12 h light/dark cycle with water and food provided ad libitum. Animals were sedated with 2% isoflurane (Belamont, Nicholas Piramal Limited, London, UK) in air. For subcutaneous tumor implantation, cells (2 × 10^6^ cells/100 µL) were injected in the leg. For brain tumor implantation, anesthetized mice were placed into a stereotaxic frame support. Cells (1 × 10^6^ or 6 × 10^5^/5 µL) were implanted in the brain—1 mm posterior, 2.5 mm lateral to the bregma, and 2.5 mm from the skull surface. The regions of the mice to be imaged were shaved with clippers and a depilatory cream before imaging.

### 4.2. Plasmid Construction

The iRFP713 cDNA was kindly provided by Dr. Vladislav V. Verskusha, Albert Einstein College of Medicine, Bronx, NY, USA [7]. The iRFP cDNA was inserted in a pcDNA5/FRT/CMV bicistronic vector containing Fluc downstream the internal ribosome entry site (IRES) of the encephalomyocarditis virus (EMCV) [16]. The iRFP cDNA were inserted downstream the CMV promoter and upstream the IRES to create a pcDNA5/FRT/CMV-iRFP-IRES-Fluc vector.

### 4.3. Cell-Line Generation and Culture

U87 cell lines (U87 MG, human glioblastoma, ATCC, Manassas, MD, USA) were cultivated in Dulbecco’s modified Eagle’s medium (Invitrogen, Carlsbad, CA, USA) complemented with 10% fetal bovine serum (Invitrogen), 1% antimycotic-antibiotic mix (PSA, Invitrogen), and 1% non-essential amino-acid (MEM NEAA, Invitrogen). Cell lines were maintained in a humidified 5% CO_2_ incubator at 37 °C. DNA construct was integrated within the genome by homologous recombination between two Flp Recombination Target (FRT) sites (Flp-in system, Invitrogen). A clonal U87-FRT cell line containing a single FRT site was used as a receiver cell line as already reported [8]. Flp recombinase, encoded in the pOG44 vector, co-transfected with DNA construct using TransFast™ Transfection Reagent (Promega, Madison, WI, USA) catalyzed recombination. Hygromycin B (50 µg/mL, Invitrogen) was used for selection of the U87-FRT-CMV-iRFP-IRES-Fluc cell line (U87-iRFP+-Fluc+). U87 cells provided by ATCC were at 150 passages, the U87-FRT clone was frozen at 170 passages, and U87-iRFP+-Fluc+ was used between 180 and 185 passages.

### 4.4. Flux Cytometry

U87-iRFP+-Fluc+ cells were harvested with trypsin and washed with PBS. For each analysis, more than 10,000 events were captured for each analysis. Excitation was performed at 640 nm, and an emission filter of 750–810 nm (APC-Cy7 filter set) was selected. Data were collected on a BD Biosciences FACSCanto apparatus (Becton Dickinson and Company, San José, CA, USA) and analyzed with FACSDiva software.

### 4.5. Bioluminescence Imaging (BLI)

BLI was performed at Vivoptic (UMS 3767—University of Bordeaux) using either a NightOWL II-LB 983 system (Berthold Technologies, Bad-Wildbad, Germany) or a Lumina LT (Perkin Elmer Inc., Boston, MA, USA). Mice received an intra-peritoneal injection of d-luciferin (Promega, 2.9 mg in 100 µL PBS) and were sedated 5 min later, and images were taken at 8 min. Brains, removed from euthanized animals approximately 20 min after d-luciferin injection, were placed in cold phosphate buffered saline (PBS) on a glass slide and imaged. For in vitro study, U87-iRFP+-Fluc+ cells were plated 48 h before imaging in 24-well culture plates. The culture medium was replaced by d-luciferin (6.10–4 M in 100 µL of PBS), and images were taken 5 min after adding the substrate. Bioluminescence images (1 min, 4 × 4 binning) and photographs (100 ms) were acquired successively. The bioluminescence signal was analyzed using IndiGO 2 software for NightOWL II-LB 983 apparatus and Living Image software for Lumina LT apparatus. 

### 4.6. Fluorescence Tomography

Mice were imaged in a fluorescent diffuse optical tomography (fDOT) prototype already described [8]. Scanning was performed using a 690 nm laser (21 mW, Power Technology, Alexander, AR, USA), and fluorescent signal filtered with a high pass RG9 filter (cut-off at 720 nm, Schott, Mainz, Germany). The transmitted images were used for the reconstruction of an optical heterogeneity map, which includes the intrinsic tissue heterogeneity and the border effects due to the complex shape of the turbid medium. The heterogeneity map was then used to reconstruct the fluorescence yield from the fluorescent images. Convergence of the algorithm was achieved within 15 iterations using a standard iterative ART algorithm with a relaxation parameter of 0.1 [18].

Mice were imaged in a Fluorescence Molecular Tomograph (FMT^®^) 4000 (Perkin Elmer Inc., Boston, MA, USA). Scanning was performed using the 670 channel, and the fluorescence signal was filtered with 690–740 nm filter emission. The images were reconstructed using the TrueQuant software (Perkin Elmer Inc., Boston, MA, USA).

### 4.7. Fluorescence Reflectance Imaging (FRI)

FRI was performed using the fDOT prototype using a light-emitting diode (LED) ring at 660 nm (Hamamatsu, Massy, France). Red fluorescence images (exposure time 1 s) were acquired through the RG9 filter with a CCD camera (Hamamatsu, Massy, France). FRI was also performed using a Lumina LT apparatus (Perkin Elmer). Excitation was performed at 675 nm, and fluorescence emission was detected with a 695–770 nm filter. For the in vitro study, U87-iRFP+-Fluc+ cells were plated in 24-well culture plates and scanned using an Odyssey scanner (Li-Core Biosciences, Lincoln, NE, USA) at 700 nm.

### 4.8. Histology and Microscopic Imaging

Brains with tumors were fixed overnight at 4 °C with 4% paraformaldehyde. The tissues were then frozen and stored at −80 °C. Brain slices (10 µm) were obtained and mounted in ProLong^®^ Gold antifade reagent with DAPI (Invitrogen). Cryosections of brain tumors were first scanned on the Odyssey scanner using channels at 700 and 800 nm. Images were then acquired with the Leica DM 5000 microscope (Leica Microsystems, Wetzlar, Germany). DAPI detection was performed using an excitation filter at 340–380 nm and an emission filter at 450–490 nm. iRFP fluorescence detection was performed using an excitation filter at 590–650 nm and an emission filter at 665–735 nm.
